# Relationship between horizontal collectivism and social network influence among college students: mediating effect of self-monitoring and moderating effect of self-efficacy

**DOI:** 10.3389/fpsyg.2024.1424223

**Published:** 2024-08-30

**Authors:** Ye Yuan, Jinchen Du, Ke Yang, Yifan Ge, Yixuan Ma, Huapei Mao, Min Xiang, Daili Wu

**Affiliations:** ^1^School of Mental Health, Wenzhou Medical University, Wenzhou, China; ^2^Zhejiang Procince Clinical Research Center for Mental Disorders, The Affiliated Wenzhou Kangning Hospital, Institute of Aging, Key Laboratory of Alzheimer’s Disease of Zhejiang Province, Wenzhou Medical University, Wenzhou, China; ^3^Department of Mathematics and Statistics, Chonnam National University, Gwangju, Republic of Korea; ^4^Sichuan Nanchong Mental Health Center, Nanchong Second People’s Hospital, Nanchong, China; ^5^School of Education Science, Guangxi Minzu University, Nanning, China; ^6^School of Medical Humanities and Management, Wenzhou Medical University, Wenzhou, China; ^7^School of Stomatology, Wenzhou Medical University, Wenzhou, China; ^8^Department of Commercial College, Wenzhou University, Wenzhou, China

**Keywords:** horizontal collectivism, self-monitoring, social network influence, self-efficacy, college students

## Abstract

**Background:**

The aim of the present study was to investigate the intrinsic relationship between cultural values and social network index among college students. In addition, the present study explored the mediating role of self-monitoring in the relationship between horizontal collectivism cultural values and social network index, as well as the moderating role of self-efficacy, to provide a theoretical approach based on the intrinsic mechanism for college students to establish a good social network.

**Methods:**

A simple random cluster sampling method was used to investigate 376 college students with cultural values scale, self-monitoring scale, self-efficacy scale, and social network index scale, structural equation model was constructed using M-plus and SPSS.

**Results:**

The result indicated that individual horizontal collectivist cultural values positively predict social network index (*β* = 0.477). Self-monitoring plays an intermediary role between cultural values and social network index, and self-efficacy plays a moderating role between self-monitoring and social network index.

**Conclusion:**

The level of an individual’s social network activity is affected by the cultural values of horizontal collectivism and self-monitoring. Improving individual self-monitoring ability and self-efficacy can effectively improve the interpersonal relationships of college students.

## 1 Introduction

With the rapid changes in social structure and cultural environment, college students, as an important force in social development, have attracted widespread attention from academia and education industry regarding their values, social behaviors, and psychological states (Yuan et al., 2022). Cultural values refer to the standards and belief systems that individuals or groups internalize to guide behavior and judgment (Schultz et al., 2022). Cultural values are one of the important factors that influence individual behavior and psychology, shaping not only worldviews and outlooks on life but also social strategies and social adaptability (Salehi et al., 2023). Cultural values mainly include collective, individual, horizontal, and vertical value orientations (Sun, 2023). Horizontal collectivism refers to the tendency in social culture for equality, sharing, and cooperation between individuals and groups, emphasizing collective harmony and consistency (Arpaci, 2019). In a culture of horizontal collectivism, individuals often perceive themselves as equal parts of others and prioritize the interests and goals of the group, providing individuals with a reliable and dependable source of information. Social Network Influence refers to the influence that an individual or group has on the behavior, attitudes, and decisions of other members in a social network. This concept not only encompasses direct interaction and communication, but also indirect influences exerted through information dissemination, emotional transmission, and social norms. Thus, horizontal collectivism maintains consistency, provides accurate information, and establishes a positive image within the group (Kaihatu et al., 2021). Trust is an important foundation for social network influence, especially in cultures that emphasize collective harmony. Horizontal collectivist values prioritize group interests over individual interests, promoting individuals to establish close social connections and a sense of community belonging, thus having a wider social network influence (Xiao, 2021).

Horizontal Collectivism is particularly evident among university students, especially regarding the influence of social networks. As a unique social group, university students’ social behaviors and information dissemination patterns are profoundly affected by cultural background, social identity, and group norms. This is mainly reflected in: (1) Equal Interaction and Emotional Support: Horizontal collectivist culture emphasizes equality and mutual aid, which is reflected in university students’ social networks as high levels of emotional support and interaction. Studies have shown that in this cultural context, university students are more likely to provide emotional support and practical help through likes, comments, and private messages. (2) Group Norms and Behavioral Consistency: group norms in a horizontal collectivist culture significantly influence university students’ behaviors. In such a culture, students are more likely to follow group behavioral norms to maintain harmony and consistency within the group. In social networks, this behavior is manifested in students’ increased participation in group activities, such as collective discussions, joint creations, and group decision-making. (3) Social Identity and Influence Diffusion: in a horizontal collectivist culture, social identity significantly affects university students’ behaviors and attitudes. Students define themselves through identification with the group and are more easily influenced by group opinion leaders. In social networks, this phenomenon is reflected in students’ tendency to follow and emulate those regarded as group representatives.

Self-monitoring refers to individuals’ ability to adjust their behavior based on social situations, emphasizing the individuals’ ability to regulate and control their behavioral performance in social interactions to achieve desired social outcomes (Fuglestad et al., 2020). The level of self-monitoring is directly related to an individual’s influence in social networks and the establishment and maintenance of interpersonal relationships, as well as the individual’s social adaptation abilities (Pillow et al., 2017). In a horizontal collectivist culture, due to the emphasis on group harmony and sharing, individuals may need to adjust their behavior and attitude more frequently to meet the needs and expectations of the collective (Leone and Yoho, 2023). Thus, horizontal collectivism may promote or require a higher level of self-monitoring ability to maintain consistency and harmony with the group. Additionally, self-monitoring ability also affects individuals’ social interaction and adaptability in a horizontal collectivist culture (Fousiani and Van Prooijen, 2023). This interaction is a two-way interaction, with cultural values and individual behavioral abilities mutually influencing and shaping each other. Under the influence of different cultural backgrounds and values, the application and effectiveness of self-monitoring may vary significantly (Hu et al., 2018). High levels of self-monitoring enable individuals to effectively manage their social behavior, thus gaining better status and influence in social networks (Bhardwaj et al., 2016). Therefore, this study hypothesis that self-monitoring may play an intermediary role between collectivist values and social network influence.

Self-efficacy is the belief in one’s ability to perform specific behaviors (Boz and Cetin-Dindar, 2023). Individuals with high self-efficacy are more likely to effectively utilize self-monitoring abilities; consequently, these individuals successfully build and maintain social networks (Chen and Ma, 2022). Therefore, this study hypothesis that self-efficacy plays a moderating role in the relationship between self-monitoring and social network influence.

At the same time, individuals with high self-efficacy may influence how they express and practice collectivist values in their social networks (Liou and Daly, 2020). Individuals with high self-efficacy may confidently promote the values of group harmony and cooperation, leading to a more positive impact within their social networks (Hu et al., 2023).

### 1.1 Hypothesis

Therefore, this study hypothesis that self-efficacy plays a moderating role in the relationship between horizontal collectivism and social network influence.

Based on the background and theoretical framework above, the present study this study proposes the following research hypotheses:

1. The level of collectivism in college students’ cultural values can positively predict individual social network influence.
2. Self-monitoring plays a mediating role between horizontal collectivism and social network influence in college students.
3. Self-efficacy plays a moderating role between self-monitoring and social network influence, as well as horizontal collectivism and social network influence.

**Figure 1:**
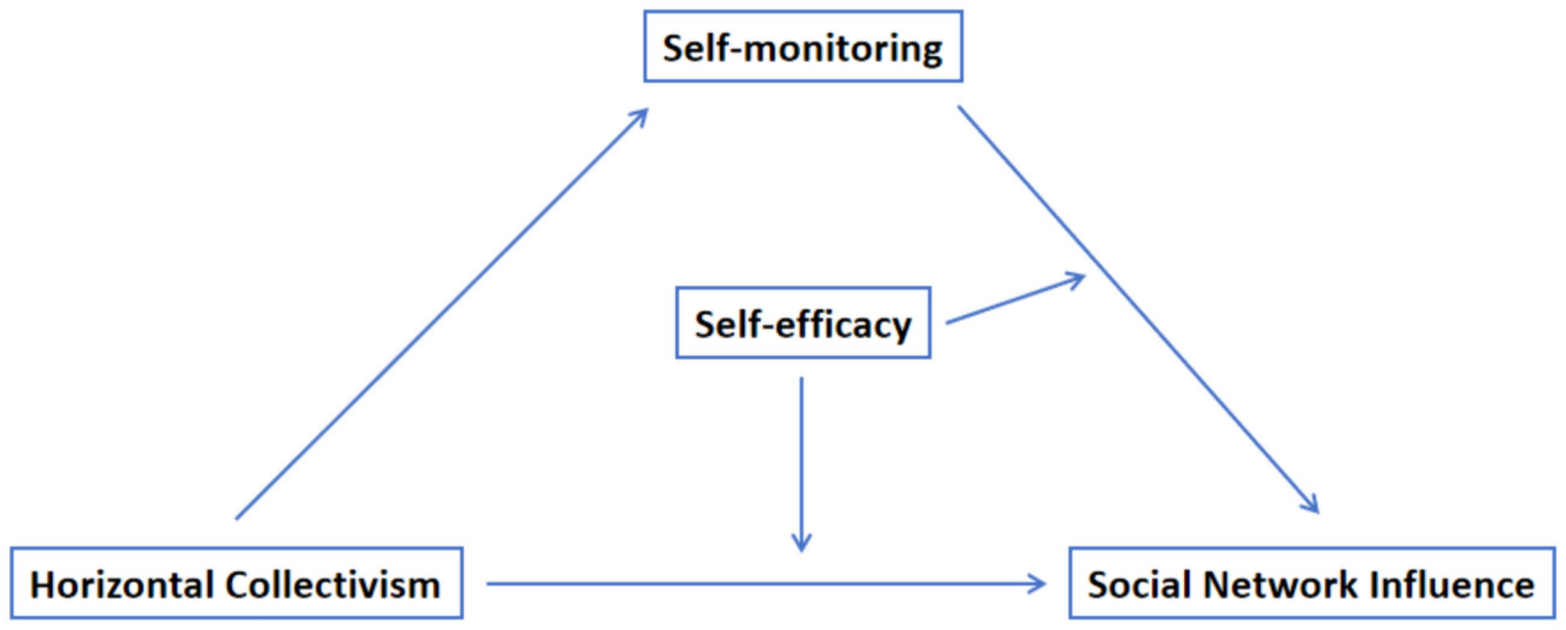
Theoretical model.

Figure 1 shows the theoretical model diagram.

The main purpose of this study is to explore the impact of horizontal collectivism on the social network of college students, and to deeply explore the mediating and moderating effects of self-management and self-efficacy, in order to provide scientific basis and guarantee for establishing healthy interpersonal relationships and promoting physical and mental health for adolescents.

## 2 Methods

### 2.1 Participants

In the present study, the research objects were full-time undergraduate students at Guangxi University and Wenzhou Medical University. A simple random cluster sampling method was used, which involved dividing the population into several groups based on a certain criterion, with each group referred to as a cluster, and then randomly sampling clusters. In the present study, random sampling was conducted at the class level. This study used a randomized method of drawing lots. Researchers can write the numbers of all groups on paper and randomly select several pieces of paper from them. For example, if there are 50 classes, you can write numbers 1 to 50 on a piece of paper, put it in a box, and then randomly select several pieces of paper. The questionnaire content was accessed via a QR code through the We-Chat Star questionnaire, researchers administered self-report measurement during regular school hours in the classroom. Before filling out the questionnaire, the researchers informed the participants about the content, purpose, and method of filling out the questionnaire. The scales followed the principles of voluntarism, anonymity, and confidentiality. Consent to use the research data was obtained from all participants. A total of 403 valid questionnaires were distributed, excluding 27 questionnaires with obvious errors or incomplete data, resulting in 376 valid questionnaires. There were 122 male students (32.4%) and 254 female students (67.6%). In addition, there were 75 students studying humanities (19.94%) and 301 students studying science and engineering (80.06%). There were 98 students in the first year of undergraduate studies (26.1%), 96 students in the second year (25.5%), 91 students in the third year (24.2%), and 91 students in the fourth year (24.2%).

### 2.2 Research measurements

#### 2.2.1 Self-monitoring scale

The Self-Monitoring Scale, developed by Snyder (1974), used to assess the degree to which individuals regulate their self-presentation and behavior in social interactions. Self-monitoring is the ability of individuals to adjust their behavior and verbal expressions according to situational changes, typically involving sensitivity to others’ reactions and self-control over their own behavior. The Self-Monitory Scale consists of 18 items in total, and the items are divided into the following five dimensions: social adaptability of self-presentation; attention to social comparison information; ability to control and modify self-presentation and expressive behavior; ability to use the aforementioned skills in specific situations; and the degree to which expressive behavior serves as a consistent or variable indicator across different contexts. The scale is scored on a binary system, with 0 indicating inaccurate description and 1 indicating accurate description. A higher score represents stronger self-monitoring ability. This scale has been effectively used in the following areas: (1) exploring self-monitoring behavior in consumers’ fashion clothing purchase decisions; (2) studying the relationship between self-monitoring and self-disclosure in intimate relationships; (3) conducting a meta-analysis on the application of self-monitoring in the workplace, evaluating the relationship between self-monitoring and factors such as job performance and leadership. In the present study, the Cronbach’s coefficient of the scale was 0.79.

#### 2.2.2 Cultural values scale

The Cultural Values Scale was developed by Choi et al. (2007). The Cultural Values Scale is primarily used to measure how individuals perceive their relationships with themselves, society, and others, and it is also utilized to elucidate how these perspectives influence their behavior, attitudes, and communication styles. The scale consists of 32 items divided into 4 dimensions, namely, horizontal individualism, horizontal collectivism, vertical individualism, and vertical collectivism. The scale uses a 7-point rating system, with scores ranging from 1 to 7, representing “strongly disagree” to “strongly agree.” Higher scores indicate a greater tendency in the respective dimensions. In the present study, the Cronbach’s *α* coefficient for the scale was 0.83.

#### 2.2.3 Social network index (SNI)

The SNI is a scale developed by Cohen in 1997 to primarily assess the breadth and depth of an individual’s social connections, including different types of social contacts, such as family, friends, and colleagues (Cohen et al., 1997). The scale consists of three dimensions, namely, network size, network diversity, and social participation. There are a total of 20 items in the scale, and the items are rated on a 5-point scale, with 1 point representing “never,” 2 points representing “rarely,” 3 points representing “sometimes,” 4 points representing “often,” and 5 points representing “always.” Higher scores indicate that an individual has a wider or denser social network, which may imply more frequent social interactions and stronger social support. In the present study, the Cronbach’s *α* coefficient for the scale was 0.83.

#### 2.2.4 General self-efficacy scale (GSES)

The GSES was developed by Schwarzer and Jerusalem (Schwarzer and Jerusalem,1995), a renowned clinical and health psychologist at Berlin University in Germany. The GSES consists of 10 items, which measure an individual’s confidence when facing setbacks or difficulties. The GSES uses a 4-point scoring system, with 1 point indicating “completely incorrect,” 2 points indicating “somewhat correct,” 3 points indicating “mostly correct,” and 4 points indicating “completely correct.” The final score is calculated by dividing the total score of the 10 questions by 10, resulting in a score range of 1–4. The critical score for the GSES is 2.5, and scores below this threshold indicate low general self-efficacy. In the present study, the test–retest reliability of this scale was 0.83, and the split-half reliability was 0.82. Moreover, the Cronbach’s *α* coefficient for the present study was 0.88.

### 2.3 Statistical processing methods

SPSS 22.0 was used to perform correlation analysis on the research data, and AMOS 21.0 and M-plus software were used to analyze the data for mediation and moderation models. A significance level of *p* < 0.05 was considered statistically significant.

## 3 Results

### 3.1 Common method deviation (CMV)

The present study adopted the self-reporting method to avoid potential CMV issues. The Harman single-factor test was used to monitor CMV, and the untwisted principal component factor test was used to statistically analyze all variables in the research data. The results revealed that the first factor explained 18.6% of the variance, which was below the critical threshold of 40%, indicating that there was no CMV issue in the research data.

### 3.2 Descriptive statistics and correlation analysis

In the present study, the horizontal collectivism values were obtained from the Cultural Value Scale. Correlation analysis showed that horizontal collectivism was positively correlated with self-monitoring, social network influence, and self-efficacy. Self-monitoring and self-efficacy were also positively correlated with social network influence. Additionally, self-efficacy was found to positively predict social network influence. Research hypothesis 1 has been validated. The analysis results are presented in Table 1.

**Table 1:** Descriptive statistical results and correlation analysis between variables.

Variables	*M* ± *SD*	1	2	3	4
1.Horizontal collectivism	41.59 ± 12.66	1			
2.Self-monitoring	12.13 ± 1.43	0.35^**^	1		
3.Social network influence	26.15 ± 7.15	0.47^**^	0.58^**^	1	
4.Self-efficacy	35.65 ± 7.68	0.28^**^	0.46^**^	0.63^**^	1

**P < 0.001.

### 3.3 Mediation effect test

First, the mediation effect of self-monitoring between horizontal collectivism and social network influence was evaluated. Second, the moderating effect of self-efficacy between self-monitoring and social network influence, as well as the moderating effect of horizontal collectivism on social network influence, were analyzed. The bias-corrected percentile bootstrap method was used to extract 5,000 samples and calculate the 95% confidence interval (CI) for the mediation moderation effect. If the confidence interval did not include a zero, it indicated a statistically significant result.

Multiple mediator analysis indicated that horizontal collectivism had a significant predictive effect on social network influence (*β* = 0.13, SE = 0.03, *t* = 4.16, *p* < 0.001, 95% CI = [0.07, 0.19]). After incorporating self-monitoring, the predictive role of horizontal collectivism on social network influence remained significant (*β* = 0.49, SE = 0.04, *t* = 13.84, *p* < 0.001, 95% CI = [0.42, 0.56]). Moreover, horizontal collectivism predicted self-monitoring (*β* = 0.22, SE = 0.03, *t* = 7.20, *p* < 0.001, 95%CI = [0.16, 0.28]), and self-monitoring played a significant role in predicting social network influence (*β* = 0.11, SE = 0.03, *t* = 3.40, *p* = 0.001, 95% CI = [0.05, 0.18]). The indirect effect these results indicated that self-monitoring mediates horizontal collectivism and social network influence. The mediating effect and corresponding effect size are shown in Table 2. The estimation of direct effect is 65% and the estimation of indirect effect is 35%, which indicate that the mediator has a huge influence between horizontal collectivism and social network influence. Research hypothesis 2 has been validated.

**Table 2:** Test of the mediation effect of self-monitoring on horizontal collectivism and social network influence.

Effect	Path	Std. estimate	Estimate	Bootstrapping 95% CI
Direct effect	Horizontal collectivism → Social network influence	0.26	65%	[−0.22, −0.11]
Indirect effect	Horizontal collectivism → Self-monitoring → Social network influence	0.14	35%	[−0.18, −0.05]
Total effect	/	0.40	100%	[−0.26, −0.18]

### 3.4 Moderation effect test

Further incorporation of self-efficacy into the model demonstrated that horizontal collectivism positively predicted social network influence (*β* = 0.39, *t* = 13.84, *p* < 0.001). The interaction of horizontal collectivism and self-efficacy had no significant effect on social network influence (*β* = 0.03, *t* = 0.22, *p* > 0.05), which indicated that the moderating effect of self-efficacy between horizontal collectivism and social network influence was not clear. The predictive role of self-monitoring on social network influence was significant (*β* = 0.48, *t* = 10.23, *p* < 0.001), and the interaction between self-monitoring and self-efficacy had a clear predictive effect on social network influence (*β* = 0.35, *t* = 9.17, *p* < 0.001). These findings indicated that self-efficacy plays a moderating role between self-monitoring and social network influence. Research hypothesis 3 has been validated. The relevant results are shown in Table 3.

**Table 3:** The mediating role analysis of self-efficacy.

Regression model		Overall fit index			Significance of regression coefficient			
Dependent variable	Predictor	*R*	*R* ^2^	*F*	*β*	LLCI	ULCI	*t*
Self-monitoring	Self-efficacy	0.39	0.14	61.65^***^	0.38	0.30	0.45	9.86^***^
Social network influence	Self-efficacy	0.54	0.29	52.13^***^	0.11	0.11	0.17	7.48^***^
	Self-monitoring				0.48	0.17	0.30	10.23^***^
	Horizontal collectivism				0.39	0.10	0.25	13.84^***^
	Self-efficacy × Horizontal collectivism				0.03	−0.23	0.08	0.22
	Self-efficacy × Self-monitoring				0.35	0.10	0.21	9.17^**^

^**^p < 0.0001, ^***^P < 0.0001.

**Figure 2:**
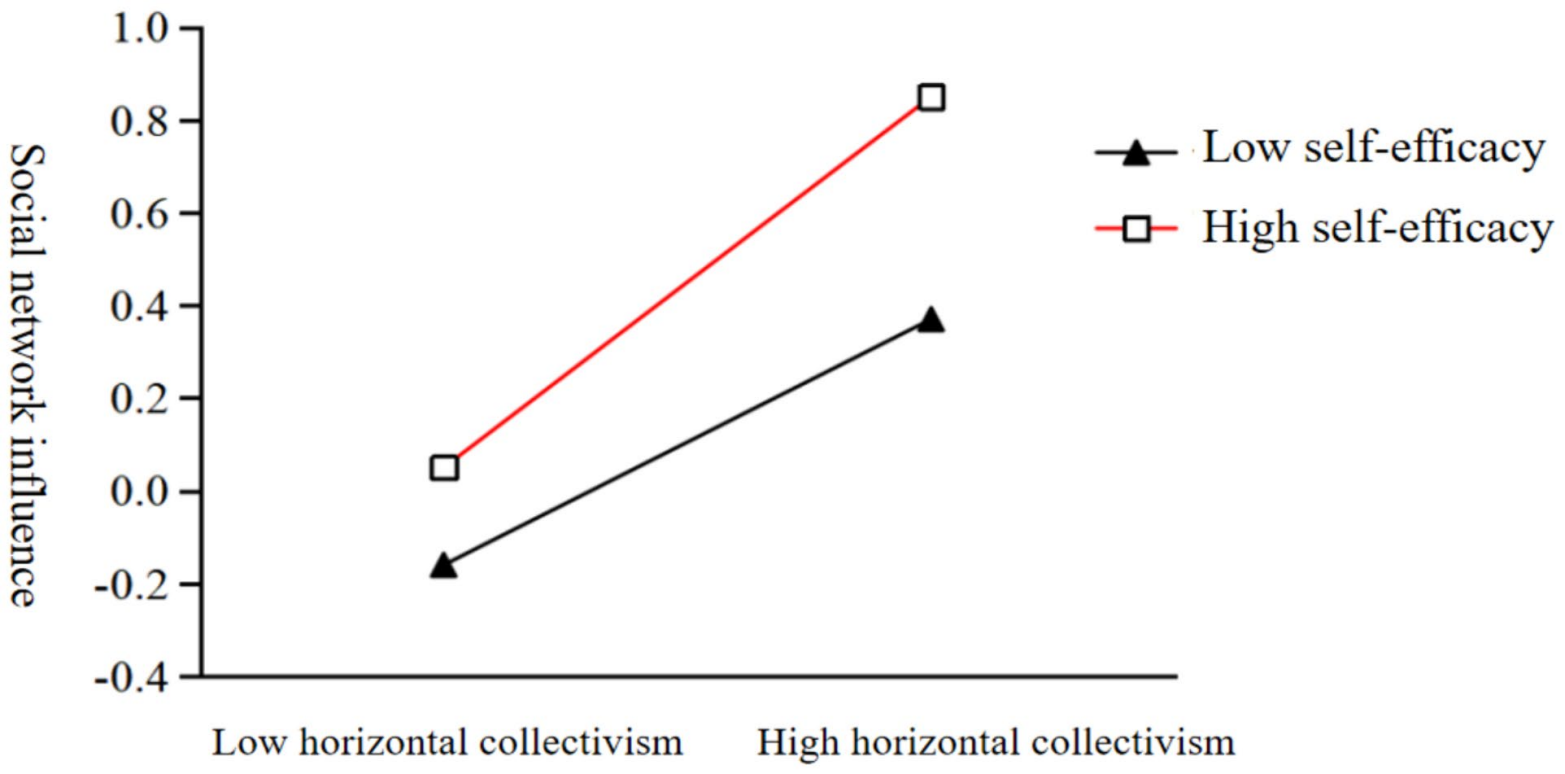
Moderating role of self-efficacy between self-monitoring and social network influence.

To examine the impact of self-monitoring on social network influence at different levels of self-efficacy, the average self-efficacy score was divided into two groups, namely, high and low, by adding and subtracting one standard deviation. Self-monitoring had a significant predictive impact on social network influence for individuals with both high and low self-efficacy (*b_simple_* = 0.48, *t* = 11.23, *p* < 0.001; *b_simple_* = 0.36, *t* = 10.73, *p* < 0.001), with higher self-efficacy individuals having a stronger predictive effect of self-monitoring on social network influence (Figure 2).

## 4 Discussion

The present study constructed a mediated moderation model, with self-monitoring as the mediator variable and self-efficacy as the moderator variable, to explore how horizontal collectivism influences the social network influence of college students. The present study demonstrated that self-monitoring is an important intrinsic factor for enhancing individual social network influence in horizontal collectivism and that this influence is affected by the moderating role of self-efficacy.

### 4.1 Relationship between horizontal collectivism and social network influence

The present study found that the level of college students’ value orientation toward horizontal collectivism was positively correlated with their social network influence, which was consistent with previous research findings that individual value differences can affect the extent of individual social interaction (Bouman et al., 2020). The Cultural Value Scale is a tool used to measure the cultural values of individuals or groups. It is often used in cross-cultural and psychological research, particularly in measuring individualism and collectivism. Harry C. Triandis’s research extensively used the Cultural Value Scale to study the manifestations of individualism and collectivism in different cultural contexts and their impact on behavior. Domestic scholars have also used the Cultural Value Scale to study the cultural values of Chinese university students (Moore, 2014). For example, some studies have analyzed the collectivist tendencies of Chinese university students through this scale, exploring their impact on learning motivation, academic achievement, and interpersonal relationships. Proponents of horizontal collectivism primarily emphasize social harmony and equal relationships with others in social interactions, pursuing common goals and interests of the group rather than emphasizing hierarchy and authority (Olsen, 2015). Individuals with a tendency toward horizontal collectivism may place greater importance on close connections with family members, friends, and community members, and they are inclined to establish and maintain extensive social networks, including within their families, among friends, and with colleagues (Dabiriyan and Yamini, 2022). At the same time, horizontal collectivists tend to maintain contact with a wide range of people and emphasize equal relationships with these connections (Booysen et al., 2021), leading to large and tightly connected social networks. Horizontal collectivists are more inclined to embrace diverse groups of people and actively maintain these relationships, thereby increasing the diversity of their social networks (To et al., 2020). Moreover, the tendency for harmony within the group may make the social networks more tightly knit and connections more frequent. Finally, horizontal collectivists not only value harmonious social relationships but also emphasize shared responsibility and support within the group (O’Connor and Gladstone, 2015). Horizontal collectivists may be more willing to provide and receive support from social networks, including emotional support, information sharing, and material assistance (Moon et al., 2018). This tendency enables horizontal collectivists to gain more resources from social networks when they are in need while also offering help to others when needed (Schermer et al., 2023). This two-way support increases the intensity and quality of social connections, thereby improving the social network index.

### 4.2 Mediating role of self-monitoring

A study by showed that using the Cultural Values Scale can assess the cultural values of South Korean university students and explore the relationship between these values and educational outcomes. The results indicated that high power distance and masculine cultural values were significantly related to students’ academic stress and mental health issues. The study suggested that university education should pay more attention to students’ cultural values and psychological needs. Analysis of the mediating effect indicated that self-monitoring plays a mediating role between horizontal collectivism and social network influence, suggesting that individuals with a tendency toward horizontal collectivism enhance their social network influence through self-monitoring. Horizontal collectivists value social harmony and the common interests of the group, suggesting that they are likely to actively use self-monitoring to maintain social relationships (Li et al., 2018; Czerniawska, 2020). Simultaneously, horizontal collectivists pay more attention to adjusting their behavior to fit the social context and the expectations of the group, thereby maintaining a harmonious social environment (Komarraju et al., 2018). In the cultural context of horizontal collectivism, the high level of self-monitoring ability can help individuals communicate and interact with others more effectively, avoiding conflicts and establishing and maintaining good social relationships (Moore, 2014). Individuals with high self-monitoring can adjust their behavior and performance flexibly in different social situations, making them more adept at establishing and maintaining social connections (Kleinbaum et al., 2015). These individuals can identify and utilize social opportunities to establish a wide range of social connections, thereby enhancing their social network influence (Bon et al., 2018). Through effective self-monitoring, individuals can better adapt to social norms, as well as attract and maintain more social connections, to ultimately form a larger, denser, and more diverse social network (Anjomshoaa et al., 2012). Therefore, enhancing college students’ self-monitoring abilities can effectively improve their social network influence and enhance their interpersonal communication skills.

### 4.3 Moderating effect of self-efficacy

The present study suggested that self-efficacy plays a moderating role between self-monitoring and social network influence. Specifically, compared to individuals with low self-efficacy, those with high self-efficacy demonstrate a stronger predictive effect of self-monitoring ability on social network influence (Rucci et al., 2021; Schueler et al., 2021). Individuals with high self-efficacy may be more effective in utilizing self-monitoring skills to enhance and expand their social networks (Siciliano, 2016). In contrast, individuals with low self-efficacy, even if they have high self-monitoring ability, may not be able to effectively improve and expand their social network because they lack the confidence and motivation to execute and maintain these social strategies (Wang et al., 2015). Thus, synchronously enhancing individuals’ self-efficacy can effectively enhance their social relationships and social network indices. The proposed moderated mediation model in the present study not only revealed the underlying mechanism of the influence of horizontal collectivism on social network influence but also explained the individual differences in this mechanism.

## 5 Limitations

There are some shortcomings in this study. First, this was a cross-sectional study exploring the impact mechanism of horizontal collectivism on social network influence in college students. Cross-sectional research has several advantages, including answering research questions and evaluating risk factors. As long as a test with high reliability and validity is selected, the results can support and explain complex models. However, cross-sectional studies also have limitations, and future research should be designed in conjunction with longitudinal follow-up studies to explore the possible causal relationship between horizontal collectivism on social network influence in college students. Second, self-reported data were used by students. Although the common method bias in this study did not reach a significant level, future research should collect data from several channels (e.g., parents, teachers, and peers) and other variables to understand the relationships between variables.

## 6 Conclusion

The present research findings suggest the importance of deepening the relationship between college students’ values and interpersonal communication, and they provide support for enhancing college students’ positive social network index. (1) The cultural value orientation of college students can influence their interpersonal relationships and social participation. Thus, actively guiding college students to embrace horizontal collectivism can effectively alleviate their social barriers and distress. (2) Implementing self-monitoring management education and guidance for college students can enhance their social enjoyment. (3) Focusing on the regulatory role of college students’ self-efficacy can improve their confidence and ability in interpersonal communication, as well as increase their social network index. Schools and teachers should offer courses or practical activities to enhance self-management among college students. By enhancing their self-management awareness and abilities, they can enhance their social skills and abilities. At the same time, they should also provide more social support to enhance their self-efficacy, enhance their social network influence, and promote healthy interpersonal relationships and physical and mental health among college students.

## Data Availability

Data will be available from the corresponding author on reasonable request.
